# Recent Insights into the Use of Antagonistic Yeasts for Sustainable Biomanagement of Postharvest Pathogenic and Mycotoxigenic Fungi in Fruits with Their Prevention Strategies against Mycotoxins

**DOI:** 10.1021/acs.jafc.3c00315

**Published:** 2023-06-23

**Authors:** Sebahat Oztekin, Dilara Nur Dikmetas, Dilara Devecioglu, Emine Gizem Acar, Funda Karbancioglu-Guler

**Affiliations:** † Istanbul Technical University Faculty of Chemical and Metallurgical Engineering Department of Food Engineering 34469 Maslak, Istanbul, Türki̇ye; ‡ Bayburt University, Faculty of Engineering Department of Food Engineering 69000 Bayburt, Türki̇ye

**Keywords:** antagonistic yeast, postharvest diseases, antifungal activity, mycotoxin, biodetoxification, biofungicide

## Abstract

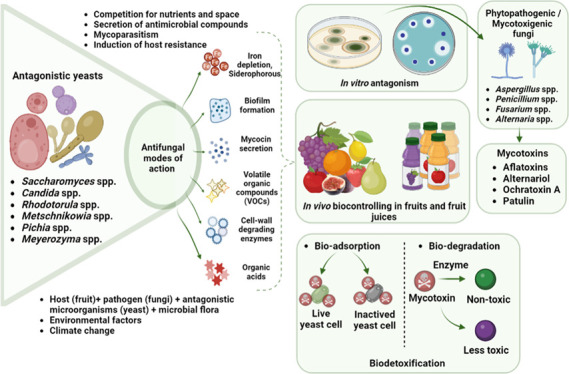

Fungi-induced postharvest diseases are the leading causes of food loss and waste. In this context, fruit decay can be directly attributed to phytopathogenic and/or mycotoxin-producing fungi. The U.N. Sustainable Development Goals aim to end hunger by 2030 by improving food security, sustainable agriculture, and food production systems. Antagonistic yeasts are one of the methods presented to achieve these goals. Unlike physical and chemical methods, harnessing antagonistic yeasts as a biological method controls the decay caused by fungi and adsorbs and/or degrades mycotoxins sustainably. Therefore, antagonistic yeasts and their antifungal mechanisms have gained importance. Additionally, mycotoxins’ biodetoxification is carried out due to the occurrence of mycotoxin-producing fungal species in fruits. Combinations with processes and agents have been investigated to increase antagonistic yeasts’ efficiency. Therefore, this review provides a comprehensive summary of studies on preventing phytopathogenic and mycotoxigenic fungi and their mycotoxins in fruits, as well as biocontrolling and biodetoxification mechanisms.

## 1 Introduction

Postharvest diseases can lead to significant food loss and waste, accounting for one-third of all food products.^1^ Among these products, fruits are highly susceptible to postharvest diseases, particularly when mechanically damaged or improperly handled. In particular, fungal postharvest diseases in fruits rank first in reducing quality and safety, thus, their shelf life.^2^ In addition to these diseases, processing fruits with postharvest diseases into new products also causes economic losses. Moreover, certain fungal species (*Aspergillus*, *Penicillium*, *Fusarium*, *Alternaria*) can produce harmful secondary metabolites called mycotoxins (e.g., aflatoxins (AF), *Alternaria* toxins, ochratoxin A (OTA), patulin (PAT), and citrinin (CIT)) in fruits. In addition, it has been stated that these toxins may occur both on the tree and during postharvest storage and are difficult to remove from processed products such as fruit juices and dried fruits.^3^ Although not entirely, they are primarily created when fungi reach maturity,^4^ and may have acute or chronic impacts on health.^5,6^ The International Agency for Research on Cancer (IARC) classifies AFB_1_ as an “extremely hazardous substance with substantial evidence of carcinogenicity in humans” (Group I), patulin and citrinin as “not carcinogenic to humans” (Group 3), OTA as a “possible human carcinogen” (Group 2B).^7−9^ It is known that there are different physical, chemical, and biological strategies to eliminate fungal-induced diseases and toxin formation.

Physical methods, including high-hydrostatic pressure, cold plasma, UV radiation, microwave, and ultrasonic treatments, require expensive special equipment and may have a negative impact on the quality of the food.^10^ However, chemical pesticides (Thiram, Amistar WG, Addstem) and some chemicals, such as sulfur dioxide (Quimetal) and ozone (Absolute Ozone, Faraday Ozone) may be preferred to inhibit phytopathogenic and/or mycotoxigenic fungi. Although these fungicides are efficient and economical, they may increase the resistance of fungal species and possible chemical residues may threaten consumer health and the environment.^11,12^ Besides, reducing pesticide use and promoting pesticide-free agriculture are among the Sustainable Development Goals (SDGs 2 and 13) of the United Nations.^13^ Biological methods have gained importance due to limitations by stricter regulatory policies and increasing consumer demand for less chemical.^14^

The use of different microorganisms to control the pathogens and diseases in agro-products^15^ and their ability to interfere with or inhibit the activity, growth, or reproduction of phytopathogens is called antagonism.^16^ Although the first reported antagonistic microorganism was a bacterium (*Bacillus subtilis*) used in the biocontrol of brown rot caused by *Monilinia fructicola* in peaches,^17^ most studies have focused on the use of antagonistic yeasts recently. Compared to bacteria and molds, yeasts have several advantages (biodegradable, genetically stable, and nonpathogenic).^18,19^ Besides, applying antagonistic yeasts as fungicides in crops leaves no hazardous residues subject to legislation. In addition to these advantages, contrary to synthetic fungicides, the application times are more flexible and can be applied to food products at the near-harvesting stage.^20^ In this regard, the European Union recently registered the active substance of *Metschnikowia fructicola* NRRL Y-27328 for use as a biofungicide,^21^ which was commercialized by Koppert Biological Systems Company under the brand name of Noli. Apart from that, there are some commercial yeast-based biofungicides, such as Excellence Bio-Nature from *Metschnikowia pulcherrima*, Boni-Protect from *Aureobasidium pullulans*, and Nexy from *Candida oleophila*.^22,23^ Commercial yeast combinations, including *M. pulcherrima* and *Torulaspora delbrueckii*, are also marketed under the trade name Zymaflore Egide (Laffort, France) for the biopreservation of juices and grapes.^24^

Microorganisms provide biocontrol not only in the postharvest period but also in the preharvest period,^25^ and preharvest application may contribute to the reduction of postharvest losses and may increase quality.^26,27^ Determination of their antifungal mechanisms (competition for space and nutrients, secretion of antimicrobial compounds, etc.) makes an important contribution to the product use. By better understanding these mechanisms, it may be possible to reformulate or discover effective biological control treatments.^28^

In addition to being effective against fungal species that cause postharvest diseases, antagonistic yeasts have been shown to be effective against mycotoxins. Among these toxins, *Alternaria* toxin generally contaminates grains, oilseeds, and fruits and vegetables such as apples, tomatoes, and citrus fruits.^29,30^*Alternaria* species can produce mycotoxins that have biological action against various metabolites, including mammals’. Alternariol (AOH), alternariol methyl ether (AME), altenuene (ALT), tentoxin (TEN), and tenuazonic acid (TeA) are examples of these metabolites.^29,31^ Aflatoxins are typically found in dry food products like cereals, spices, and dried fruits.^32^ AFB_1_ contamination has been reported on dried fruits, including figs, raisins, currants, sultanas, plums, dates, and apricots, which have the lowest frequency (36%) of food products.^33^ Physical, biological, or chemical methods together with antagonistic yeasts might be alternatives to eliminate or avoid aflatoxin contamination in fruits. CIT is a potent nephrotoxin, especially produced by the *Monascus*, *Penicillium*, and *Aspergillus* sp.^34^ CIT contamination has been reported in different fruits and vegetables at different locations such as apples, cherries, figs, pears, grapes, and fruit juices.^35,36^ Various fungal species from the genera of *Penicillium*, *Aspergillus*, and *Byssochlamys* generate the poisonous secondary metabolite PAT, which is frequently found in apple products.^37,38^ The current studies addressed the susceptibility of grapes to *Aspergillus* and *Penicillium* species found in wine, dried grapes, and grape juice contaminated with ochratoxin A.^39,40^ PAT in apples and OTA in grapes are two mycotoxins that are currently the focus of research in fruits, whereas mycotoxins in citrus fruits are rarely observed. Additionally, mycotoxin contamination in fruits and fruit products has been reported in several studies (Table 1**)**.

**Table 1:** Occurrence of Mycotoxins in Fruits and Fruit Products around the World

mycotoxin	commodity contaminated	country	references
aflatoxin	fruit juices (apple, orange, pear, pineapple)	Spain	(242)
	dried fruits (mulberry, date, figs, apricot)	Pakistan	(243)
	fig jam, biscuit with fruit filling	Serbia	(244)
	dried figs	Türkiye	(245)
	dried fruits	Iran	(246)
alternaria	dried figs	Netherland	(247)
	apple juice	China	(248)
	apple, apricot, citrus and grape juice	Germany	(249)
	jujube	China	(250)
citrinin	apples	Portugal	(251)
		China	(36)
		Crotia	(36)
	grape	Czech Republic	(38)
patulin	grape	Czech Republic	(38)
	grape, apricot, peach, pear	Argentina	(252)
	apple juice, pear juice	Tunisia	(9)
	apple juice, apple and pear concentrates	Spain	(253)
	apple, orange, mango, lemon juices	China	(254)
	apple	Pakistan, Canada, Thailand, Qatar	(255)
	apple juice	China	(256)
	grape, pear, peach	Argentina	(252)
	strawberry	Poland	(257)
	apple juice, pear juice, strawberry juice	Czech	(258)
	apple sour	Türkiye	(259)
ochratoxin A	grape juice	Türkiye	(260)
	grape juice	China	(261)
	fruit juice	Argentina	(252)
	raisin	Pakistan	(262,263)
	dried grape, grape juice	Iran	(246,264)

Removal or control of these toxins by using antagonistic yeasts is presented as a cost-effective, environmentally friendly, and safe application.^41^ Therefore, yeast-based detoxification methods have captured great interest as a green, affordable, specific, and effective approach for removing mycotoxins from fruit-related environments.^42^ In this regard, yeast cells and their biodegrading enzymes effectively absorb and transform mycotoxins in fruits and fruit-based products (PAT, OTA, AF, CIT, and *Alternaria* toxins) into non- or less-toxic compounds, but sometimes into highly toxic derivatives.^43,44^ However, biodetoxification may require antagonistic yeasts for degradation and adsorption.

As mentioned, it is important to increase the activity of antagonistic yeasts, and at this point, combination studies with different processes and agents for fruits are seen. In the current study, the use of antagonistic yeasts as biocontrol agents against plant-pathogenic fungi and their mycotoxins in fruits was critically reviewed by covering antifungal mechanisms of action, biocontrol and mycotoxin prevention strategies, and the combined application of yeasts for enhanced biocontrol activity. This review will assist scientists in understanding how antagonistic yeasts can be used as a safe and sustainable solution for the security and safety of fruits and fruit products, including juices and dried fruit.

## 2 Antagonistic Yeasts and Their Antifungal Mechanisms of Action

### 2.1 Features of Antagonistic Yeasts

Although the first reported antagonistic microorganism used in biological control was a bacteria, it has been suggested that using antagonistic yeasts is more advantageous in preventing postharvest diseases.^45^ The yeasts whose antagonistic activity is studied mostly belong to the genera of *Metschnikowia*, *Debaryomyces*, *Aureobasidium*, *Saccharomyce*s, *Candida*, *Pichia*, and *Meyerozyma.*(46−48) The important superiorities of yeast antagonists over bacteria or mycelial fungi are (i) yeasts are more resistant to stress factors, even to UV, (ii) yeasts can develop adequately in fruits despite their high sugar content and acidic structure, (iii) yeasts can easily colonize even dry surfaces by the usage of inexpensive and simple substrates, and (iv) yeasts do not produce toxic secondary metabolites or allergenic spores.^14,28,49,50^ Although mostly the yeasts that naturally occur on the surfaces of fruits are used in the biocontrol of postharvest diseases,^14^ there are also antagonistic yeasts isolated from animal ecosystems,^51^ Blue-veined Rokpol cheese,^52^ water samples collected from Antarctica,^53^ soil from King George Island (Antarctica),^54^ and terrestrial of King George Island.^55^ The growth characteristics may change according to the environment in which the yeast is isolated. For instance, the resistance to extreme conditions, including high osmotic pressure and abiotic stress, is higher in yeasts isolated from marine rather than from fruit surfaces.^56^

Factors such as growth conditions, nutrient requirements, simplicity, activity range, effectiveness, and safety should be considered when deciding which antagonistic yeast to use from a wide range, such as the source of isolation and the genera it belongs to.^19,57^ According to Wilson and Wisniewski,^58^ antagonistic yeasts should be genetically stable, effective at low concentrations and against various pathogens, resistant to the chemicals used, able to tolerate extreme conditions, easy to dispense, adaptable to processing techniques, not harmful to humans, and have long shelf life. Additionally, the microorganisms used in food must be on the GRAS list approved by the FDA because of the necessary toxicity tests.^59^ There are GRAS reports of various yeast genera that declare that there is no health risk for the consumption of food that contacts these microorganisms. Yeasts that can be used in the biocontrol of the diseases caused by the fungal pathogens in fruits should be selected considering these criteria.

Antagonistic yeasts can follow different pathways while inhibiting the growth and pathogenicity of fungal pathogens. It is critical to choose the optimum antagonistic yeast and ambient conditions to prevent postharvest disease in fruits and understand the mechanism that underlies the antagonistic effect to provide effective biological control. There is a continuous interaction between the antagonistic yeast, the fungal pathogen, the host fruit, and the natural microbiota of the host fruit. It is observed that yeasts in the pseudohyphal form may damage the tissues and accelerate the deterioration of a host fruit, while it has a successful antagonistic effect on another fruit.^60^ Therefore, yeast’s antagonistic properties should be tested for each host fruit. This complex relationship is highly influential on the mechanism of action of each antagonist, and the situation should be thoroughly studied to understand the mechanism.^14,28^

### 2.2 Antagonistic Yeasts’ Antifungal Mechanisms of Action

**Figure 1:**
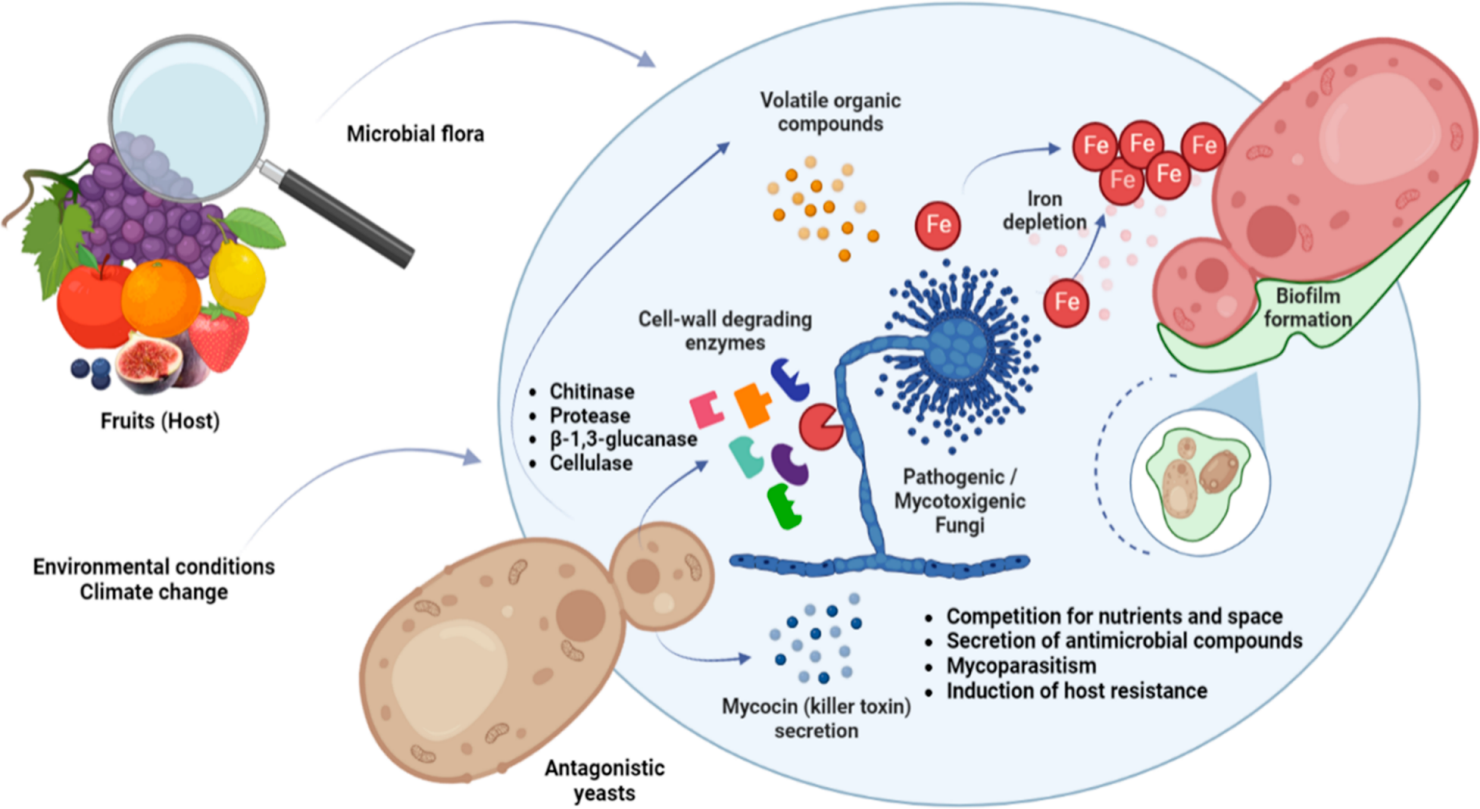
Antifungal mechanisms of action of antagonistic yeasts.

The main antifungal mechanisms of antagonistic yeasts are competition for nutrients and space, secretion of antimicrobial compounds, enzyme-related mechanisms, mycoparasitism, and induction of host defense.^61^ The main antifungal mechanisms of action and factors are summarized in Figure 1. Antagonistic modes of action can be both direct and indirect. Direct mechanisms are highly selective for fungal pathogens; in contrast, indirect mechanisms are not specific to the pathogen. While the competition for nutrients and space, secretion of antimicrobial compounds, and mycoparasitism are direct mechanisms, induction of host defense is an indirect antagonistic mechanism.^15,62^ In vitro and *in vivo* recent studies on the mechanisms of antagonistic yeasts against fungal pathogens are summarized in Tables 2 and 3.

**Table 2:** Representative *In Vitro* Antifungal Mechanisms Of Action of Antagonistic Yeasts Used in Recent Years

action mechanism	antagonistic yeast	origin	fungal pathogen	reference
competition for nutrient and space	*Candida stellimalicola* ACBL-07	citrus leaves	*Penicillium italicum*	(265)
	*Debaryomyces hansenii* ECP4	surface of papaya	*Colletotrichum gloeosporioides*	(84)
	*Debaryomyces nepalensis*	soil of unsprayed mango orchards	*Colletotrichum gloeosporioides*	(266)
	*Pichia kudriavzevii* S_2_C	kimchi	*Penicillium digitatum*	(70)
	*Metschnikowia citriensis* FL01	citrus leaves	*Geotrichum citri-aurantii*	(105)
	*Metschnikowia pulcherrima* 26-BMD	blackberry	*Penicillium digitatum, Penicillium expansum*	(86)
biofilm formation	*Candida oleophila* L12, *Debaryomyces hansenii* L16	fruits of several citrus varieties	N/A^a^	(267)
	*Cryptococcus laurentii* FRUC DJ1	grapefruit	N/A	(268)
	*Debaryomyces nepalensis*	soil of unsprayed mango orchards	N/A	(266)
	*Metschnikowia* aff. *fructicola* 1-UDM, *Metschnikowia pulcherrima* 26-BMD	green grapes blackberry	N/A	(86)
	*Pichia kudriavzevii* S_2_C	kimchi	N/A	(70)
	*Torulaspora indica* DMKU-RP31	leaves of economic plants	N/A	(269)
	*Vishniacozyma victoriae* EPL4.5 and EPL29	apple		(270)
volatile organic compounds (VOCs)	*Aureobasidium pullulans* GE17, *Meyerozyma guilliermondii* KL3	Golden Delicious apple	b	(271)
		Kutdiken lemon		
	*Candida oleophila* L12, *Debaryomyces hansenii* L16	fruits of several citrus varieties	*Penicillium digitatum, Penicillium italicum*	(267)
	*Candida sake* 41E	soil and water samples collected in antarctica	*Penicillium expansum*	(53)
	*Debaryomyces hansenii* ECP4	surface of papaya	*Colletotrichum gloeosporioides*	(84)
	*Galactomyces geotrichum* JYC549	cherry tomato	*Fusarium proliferatum*	(272)
	*Metschnikowia pulcherrima*	soil of a mango	*Colletotrichum gloeosporioides*	(273)
		orchard		
	*Metschnikowia pulcherrima* 34-UEM	red grapes	*Penicillium digitatum*, *Penicillium expansum*	(86)
	*Meyerozyma guilliermondii/M. caribbica*, *Pichia occidentalis*, *Pichia kudriavzevii/Issatchenkia orientalis*	organic grape	*Penicillium chrysogenum*, *Botrytis cinerea*	(80)
	*Pichia galeiformis* BAF03	lemon	*Penicillium digitatum*	(274)
	*Wickerhamomyces anomalus* BS91	naturally fermented olive brine	*Monilinia fructigena* and *Monilinia fructicola*	(52)
killer toxin	*Candida stellimalicola* ACBL-07	citrus leaves	*Penicillium italicum*	(265)
	*Debaryomyces hansenii* MI1a and KI2a	blue-veined Rokpol cheese		(52)
secretion of lytic enzyme	*Aureobasidium pullulans* GE17, *Meyerozyma guilliermondii* KL3	Golden Delicious apple	N/A	(271)
		Kutdiken lemon	N/A	
	*Candida oleophila* L12, *Debaryomyces hansenii* L16	fruits of several citrus varieties	N/A	(267)
	*Debaryomyces hansenii* ECP4	surface of papaya	N/A	(84)
	*Debaryomyces hansenii* MI1a and KI2a	blue-veined Rokpol cheese	N/A	(52)
	*Pichia galeiformis* BAF03	lemon	N/A	(274)
	*Saccharomyces cerevisiae* ACBL-11	citrus flower	N/A	(265)
adhesion to fungal hyphae (mycoparasitism)	*Debaryomyces nepalensis*	soil of unsprayed mango	*Colletotrichum gloeosporioides*	(266)
		orchards		
	*Metschnikowia pulcherrima* L672	fig	*Cladosporium cladosporioides*	(275)
	*Pichia kudriavzevii* S_2_C, *Yarrowia lipolytica* S_4_A	kimchi	*Penicillium digitatum*	(70)

a Not applicable.

b It is not specified.

**Table 3:** Representative *In Vivo* Antifungal Mechanisms of Action of Antagonistic Yeasts Used in Recent Years

action mechanism	antagonistic yeast	origin	fungal pathogen	host fruit	reference
competition for nutrient and space	*Hanseniaspora opuntiae* M479, *Metschnikowia pulcherrima* L672	fig	*Penicillium expansum*, *Botrytis cinerea*, *Cladosporium cladosporioides*	apple	(274,275)
	*Metschnikowia pulcherrima*	soil of a mango orchard	*Colletotrichum gloeosporioides*	mango	(273)
	*Meyerozyma guilliermondii*	surface of rotten pears	*Penicillium expansum*	pear	(276)
	*Metschnikowia* aff. *fructicola* 1-UDM	green grapes	*Penicillium digitatum*, *Penicillium expansum*	lemon	(86)
wound site colonization	*Candida oleophila* P316	surface of pear fruit	N/A^a^	kiwifruit	(277)
	*Meyerozyma guilliermondii*	surface of rotten pears	N/A	pear	(276)
	*Meyerozyma guilliermondii* Y-1	grape	N/A	apple	(196,278)
	*Pichia galeiformis* BAF03	lemon	N/A	citrus fruit	(274)
	*Wickerhamomyces anomalus* BS91	naturally fermented olive brine	N/A	peach and plum	(52)
adhesion to fungal hyphae (mycoparasitism)	*Cryptococcus laurentii* FRUC DJ1	grapefruit	*Penicillium digitatum*	grapefruit	(268)
	*Debaryomyces nepalensis*	soil of unsprayed mango orchards	*Colletotrichum gloeosporioides*	mango	(266)
	*Meyerozyma caribbica*	b	*Colletotrichum gloeosporioides*	mango	(88)
volatile organic compounds (VOCs)	*Candida sake* 41E	soil and water samples collected in Antarctica	*Penicillium expansum*	apple	(53)
	*Hanseniaspora uvarum* 793	fig	*Botrytis cinerea*	strawberries and cherries	(279)
	*Wickerhamomyces anomalus*, *Metschnikowia pulcherrima*, *Saccharomyces cerevisiae*		*Botrytis cinerea*, *Monilinia fructicola*, *Alternaria alternata*, *Aspergillus carbonarius*, *Penicillium digitatum*, *Cladosporium* spp., *Colletotrichum* spp.,	strawberry	(280)
	*Torulaspora indica* DMKU-RP31	leaves of economic plants	*Lasiodiplodia theobromae*	mango	(269)
secretion of defense-related enzyme	*Candida oleophila* P316	surface of pear fruit	N/A	kiwifruit	(277)
	*Cryptococcus laurentii* FRUC DJ1	grapefruit	N/A	grapefruit	(268)
	*Debaryomyces hansenii* AII4b	blue-veined Rokpol cheese	*Monilinia fructicola*	apple	(125)
	*Debaryomyces nepalensis*	soil of unsprayed mango orchards	*Colletotrichum gloeosporioides*	mango	(266)
	*Kluyveromyces marxianus* XZ1	apple	N/A	apple	(281)
	*Metschnikowia pulcherrima*	soil of a mango orchard	*Colletotrichum gloeosporioides*	mango	(273)
	*Meyerozyma guilliermondii*	surface of rotten pears	N/A	pear	(276)
	*Meyerozyma guilliermondii* Y-1	grape	N/A	apple	(196,278)
	*Pichia guilliermondii*		N/A	peach	(192)
induction of host defense	*Candida oleophila* P316	surface of pear fruit	N/A	kiwifruit	(277)
	*Kluyveromyces marxianus* XZ1	apple	N/A	apple	(281)
	*Meyerozyma guilliermondii*	surface of rotten pears	N/A	pear	(276)
	*Meyerozyma guilliermondii* Y-1	grape	N/A	apple	(196,278)
	*Pichia guilliermondii*	N/A	N/A	peach	(192)

a Not applicable.

b It is not specified.

#### 2.2.1 Competition for Nutrients and Space

Competition for nutrients and space is the main mechanism of action since all antagonists can exert this mechanism to some extent,^63^ and the antagonists invade host fruits by growing in wounds from the very first contact and causing depletion by consuming nutrients.^19^ For the competition for space, antagonistic yeasts should be able to colonize on the host fruit surface, and biofilm formation can enhance the colonization.^64^ Therefore, the superiorities of antagonistic yeasts acting with this mechanism are the abilities to grow fast and form biofilms through the wounded fruit surface.^28^ The type and natural microbiota of the host fruit and the concentration of antagonistic yeast can change these properties.^65^ Yeasts communicate about their cell density and express genes by quorum sensing for biofilm formation.^66^ Biofilms are formed by the aggregation and adhesion of yeast cells to themselves and the surface, the formation of an extracellular matrix composed of DNA, exopolysaccharides and proteins, and proliferation.^67^ Antagonistic yeasts can tolerate stress conditions better in the form of biofilm.^67,68^ In one study, it was observed that the transition of *Pichia kudriavzevii* from the yeast-like form to the biofilm form increased its tolerance to heat and oxidative stress and significantly reduced the lesion diameters and disease incidence caused by *Botrytis cinerea* and *Colletotrichum gloeosporioides* in pear.^69^

For the competition for nutrients, antagonistic yeasts consume the nutrients in host fruits, including carbon and nitrogen, causing pathogenic fungi to be unable to reach essential nutrients for viability and growth.^62^ It is stated that when sufficient micronutrients, nitrogen and carbon sources are provided, a significant reduction is seen in the inhibition rate of *Penicillium digitatum* germination by the *Pichia kudriavzevii*.^70^ Another component, iron, can act as a limiting factor for fungal growth since it is present in various proteins and enzyme structures. Antagonistic yeast can produce iron chelators and siderophores to compete for iron. These mechanisms cause iron depletion and inhibit the conidial germination and pathogenesis of fungi.^28^ Some antagonistic yeasts secrete pulcherriminic acid, which reacts with Fe^3+^ and produces an iron chelate called pulcherrimin.^23^ Pulcherrimin is an insoluble red pigment whose intensity increases with the amount of depleting iron in the medium and is correlated with the antagonistic activity of *Metschnikowia pulcherrima*.^71,72^

#### 2.2.2 Secretion of Antimicrobial Compounds

Antagonistic yeasts can produce antimicrobial molecules as secondary metabolites, which are organic molecules that inhibit other microorganisms due to their toxicity.^62^ The possibility of the developing resistance in pathogens to antimicrobials produced by antagonists and health concerns should be considered.^28^ Killer toxins and VOCs are antagonistic yeasts’ main antifungal secondary metabolites. Killer toxins, also called mycocins, are protein, glycoprotein, or glycolipids with a molecular weight of 10–30 kDa,^73^ produced for the competition with other environmental microorganisms and increasement of stress resistance.^74^ Toxin-producer yeast can compete for nutrients and space in the host fruit since the killer toxin secreted by the antagonistic yeast does not affect itself; it only has a lethal effect on other yeasts and molds.^68,75^ The action mechanisms of killer toxins include disrupting the cell membrane, inhibiting cell wall and DNA synthesis, and blocking calcium uptake.^73,75,76^ Mycocins are advantageous biocontrol mechanisms due to their nontoxicity to mammals, increased resistance to stress conditions, environmental friendliness, and decreased possibility of pathogen resistance.^19^ A study showed that the killer toxins of *Debaryomyces hansenii* strains were highly stable at pH 2.5–5.5 and 5–37 °C and could inhibit *Alternaria brassicicola*, *Alternaria citri*, *Aspergillus niger*, and *Rhizopus stolonifer* in fruits.^77^ Grzegorczyk et al.^52^ stated that the killer toxin activities of two different strains might vary because of protein structure and enzymatic activity changes.

Other antifungal secondary metabolites, volatile organic compounds (VOCs), are highly water-soluble molecules with high vapor pressure and smaller than 300 Da, which may include aldehydes, ketones, hydrocarbons, alcohols, thioalcohols, cyclohexanes, heterocyclic compounds, phenols, etc.^46^ A study conducted with *Wickerhamomyces anomalus* BS91, *Metschnikowia pulcherrima* MPR3, *Aureobasidium pullulans* PI1, and *Saccharomyces cerevisiae* BCA61, the main VOCs produced by antagonist yeasts were found to contain ethyl alcohol, ethyl acetate, isoamyl alcohol, isoamyl acetate, and phenethyl alcohol.^78^ The composition of VOCs may vary according to the producer antagonist yeast, fungal pathogen, and the ecological niche,^79^ thus can be defined as “strain and target-dependent.”^23^ When the *in vitro* effect of VOCs of four different yeasts (*Pichia kudriavzevii* KKP 3005, *Pichia occidentalis* KKP 3004, *Meyerozyma guilliermondii, Meyerozyma caribbica* KKP 3003) against five different fungal pathogens belonging to *Penicillium*, *Fusarium*, *Aspergillus*, *Mucor* and *Botrytis* was examined, it was observed that the composition of the VOCs secreted by the same yeast species was changed in the headspace in contact with different fungal pathogens.^80^ Similarly, when the VOCs of three different isolates belonging to *Saccharomyces cerevisiae* were examined, the gas composition changed, even though the most abundant molecules were the same.^81^ Their action mechanism is based on the modification of amino acid, protein and nucleus synthesis, and inhibition of the fungal pathogen.^76^ Studies on VOCs produced by antagonists have recently intensified besides using antagonistic yeasts in biocontrol. Outstanding advantages such as being biodegradable, being effective even in small amounts,^82^ not requiring direct contact between antagonistic yeast and pathogen, and being able to spread rapidly by gas diffusion in an inhomogeneous medium consisting of solid, liquid, and gas, where the target is far away, have made the VOC mechanism very promising and popular.^80^ The most important issue is that the VOCs’ composition and fungal inhibition rates may vary with the *in vitro* results and in the natural conditions with the host fruit.^28^

#### 2.2.3 Mycoparasitism

Mycoparasitism is the mechanism of action in which antagonistic yeast is attached to the hyphae of the fungal pathogen and secretes cell wall-degrading enzymes that cause fungal lysis or destruction.^63^ While glucan, the main structural polysaccharide that acts as a filling material, makes up 50–60% of the cell wall, the other 40–50% consists of half chitin, the backbone of the cell wall, and half of the protein.^28,83^ Since yeasts disintegrate fungal cell walls to reach carbon sources and amino acids for their viability,^84^ mostly, activities of β-1,3-glucanase (GLU), Chitinase (CHT), and proteases are strongly associated with the antagonistic activity.^61,85^ Similar to other metabolites, enzyme profiles of antagonistic yeasts may also differentiate. Oztekin and Karbancioglu-Guler^86^ examined the protease, pectinase, cellulase, GLU, and gelatinase enzyme activities of different *Metschnikowia pulcherrima* isolates. They reported that an isolate produced all enzymes, one isolate did not produce pectinase, and one isolate did not produce protease.

The first evidence of mycoparasitism was the attachment of *Pichia guilliermondii* to fungal hyphae and the secretion of GLU against *B. cinerea*.^87^ More recently, the adhesion of *Meyerozyma caribbica* to the hyphae of the fungal pathogen *Colletotrichum gloeosporioides* on mango evidenced the action mechanism of mycoparasitism.^88^

#### 2.2.4 Induction of Host Defense

Unlike the mechanisms described above, the induction of host defense is an indirect mechanism; that is, the antagonistic yeast does not directly affect the pathogenic fungi but increases the resistance of the host fruit of the fungi against this pathogen.^62^ Pathogens and environmental factors also affect how antagonistic yeasts induce resistance,^19^ and the induction of host resistance may occur by different mechanisms, but briefly, antagonistic yeasts stimulate the defense signals through elicitor, the expression of antioxidant genes and defense-related enzymes, and the production of reactive oxygen species (ROS), especially H_2_O_2_, in the host fruit.^89−91^ The secretion of both pathogenesis-related (PR) proteins (GLU and CHT) and defense-related or antioxidant enzymes [phenylalanine ammonia-lyase (PAL), peroxidase (POD), polyphenol oxidase (PPO), and catalase (CAT) etc.] by antagonistic yeasts may induce resistance in the host fruit.^61,92^

Cheng et al.^93^ showed that *Hanseniaspora uvarum* induces host defense of kiwifruit by activating defense-related genes and enzymes, CHT and GLU. Another antagonist yeast, *Metschnikowia pulcherrima* E1, could induce host resistance in loquat fruit to *Pestalotiopsis vismiae* by enhancing the activities of defense-related enzymes of PAL, POD, PPO, CAT, and ascorbate peroxidase (APX).^94^ The application of *Rhodotorula mucilaginosa* on strawberries has delayed the senescence and induced the host resistance by promoting both the defense-related enzymes and PR proteins against *Rhizopus stolonifer* and *Botrytis cinerea.*(95) It has also been reported that the induction of host defense by the biosynthesis of phenylpropanoid is the action mechanism of *Pichia galeiformis* against *Penicillium digitatum* on citrus fruit.^96^ Proteomic studies should be increased to understand this mechanism better, explore the plant’s affected metabolic pathways, and find the most effective antagonist against fungi.

## 3 Antagonistic Yeasts As Biocontrol Agents

In developed countries, the predicted postharvest losses reached 25% of total production, while in undeveloped countries, they were greater than 50%.^97^ The Food and Agriculture Organization reported that plant diseases cost 220 billion annually.^98^ Fungal or fungus-like microorganisms have caused about 85% of postharvest diseases in fruits.^99^ It is well documented that most of the fruit losses at the postharvest stage is highly caused by fungal pathogens such as *Alternaria*, *Aspergillus*, *Botrytis*, *Colletotrichum*, *Diplodia*, *Monilinia*, *Penicillium*, *Phomopsis*, *Rhizopus*, *Mucor*, and *Sclerotinia*.^100^ Postharvest diseases by fungal pathogens have been summarized as brown rot, blue mold, green mold, gray mold and anthracnose caused by *Monilinia* sp., *Penicillium expansum*, *Penicillium digitatum*, *Botrytis cinerea*, and *Colletotrichum musae*, respectively.^101^ Fruits with those diseases might pose a health risk, in addition to economic problems, since several fungal species including *Penicillium*, *Alternaria*, and *Fusarium* produce mycotoxins that are dangerous to people’s health.^14^ Numerous studies have reported biological control methods with several bacteria, yeasts and filamentous fungi, which are eco-friendly alternatives for controlling postharvest fruit losses against synthetic fungicides.^14,95,96,102−106^ Most postharvest decays in fresh fruits are caused by the fungi *Penicillium* and *Botrytis*.^100^ Due to their wide host range, multiple attack strategies, and being in asexual and sexual phases give them the capacity to live both in favorable or unfavorable environments, these pathogens continue to be challenging to manage.^107^

### 3.1 Management of Postharvest Fungal Diseases by Antagonistic Yeasts

A potential option for fruit protection against phytopathogens at the postharvest stage is presently provided by biocontrol strategies, which protect plants against fungal infections.^101^ Various yeast species have been tested for their ability to prevent postharvest fruit diseases with *in vitro* and *in vivo* studies (Tables 4 and 5).

**Table 4:** Evaluation of the Antifungal Activity of the Yeast with *In Vitro* Studies

yeast strain	origin	target fungi	antagonistic assays	optimum concentration of biocontrol agent	inhibition of mycelial growth (%)	references
*Aeorobasidium pullulans*	grape berries	*Aspergillus tubingensis*	dual culture method	a	12.97–95.69	(282)
*Candida catenulata*	fruits and leaves of citrus plants	*Penicillium digitatum*	dual culture method		<15	(50)
*Candida tropicalis*	mandarin orange	*Colletotrichum musae*	dual culture method	1 × 10^8^ cells/mL	31.3	(283)
*Debaryomyces hansenii*	citrus varieties	*Penicillium digitatum Penicillium italicum*	dual culture method		41	(267)
					6	
*Debaryomyces hansenii*	marine environment	*Mucor circinelloides*, *Aspergillus* sp., *Fusarium proliferatum*, *Fusarium subglutinans*	agar plate inhibition	1 × 10^8^ cells/mL	97.2–98.3	(284)
*Debaryomyces hansenii*	blue-veined Rokpol cheese	*Monilinia fructicola*	dual culture method	1 × 10^8^ cells/mL	69.53	(125)
*Hannaella pagnoccae*, *Hannaella luteola*, *Vishniacozyma carnescens*, *Hannaella* spp., *Rhodotorula paludigena*	cacao pods and leaves	*Moniliophthora roreri*	dual culture method		>71	(285)
*Hanseniaspora opuntiae*		*Penicillium expansum*, *Botrytis cinerea*	dual culture method	1 × 10^6^ cells/mL	45.2	(275)
					53.4	
*Metschnikowia* aff. *pulcherrima*	hawthorn	*Penicillium expansum*, *Penicillium digitatum*	dual culture method	1 × 10^8^ cells/mL	84.2	(86)
					95.3	
*Pichia fermentans*	fruits and leaves of citrus plants	*Penicillium digitatum*	dual culture method		16–39	(50)
*Pichia fermentans*	grapes	*Penicillium expansum Botrytis cinerea*	dual culture method	1 × 10^6^ cells/mL	0–25	(286)
*Pichia kluyveri*	marula juice	*Botrytis cinerea Monilinia laxa*	agar plate inhibition	1 × 10^8^ cells/mL	44.5	(287)
					54.6	
*Rhodoturula mucilaginosa*	black olives	*Aspergillus carbonarius*	disc diffusion method	1 × 10^5^ cells/mL	75.8	(141)
*Rhodoturula glutinis*	apple	*Monilinia fructigena*	dual culture method		18	(288)
*Rhodotorula paludigena*	cacao pods and leaves	*Moniliophthora roreri*	dual culture method		>71	(285)
*Saccharomyces cerevisiae*	fruits and leaves of citrus plants	*Penicillium digitatum*	dual culture method		16–39	(50)
*Saccharomyces cerevisiae*	black olives	*Aspergillus carbonarius*	disc diffusion method	1 × 10^5^ cells/mL	66.1	(141)
*Saccharomyces cerevisiae*	citrus	*Penicillium italicum*	pour plate method	1 × 10^7^ cells/mL	>80	(265)
*Saccharomyces cerevisiae*	mango	*Colletotrichum musae*	dual culture method	1 × 10^8^ cells/mL	24.3	(283)
*Saturnispora diversa*	loquat	*Colletotrichum gloeosporioides*		1 × 10^7^ cells/mL	74–85.7	(289)
*Wickerhamomyces anomalus*	grapes	*Penicillium expansum*, *Botrytis cinerea*	dual culture method	1 × 10^6^ cells/mL	76–100	(286)
*Wickerhamomyces anomalus*	blue-veined Rokpol cheese	*Monilinia fructicola*	dual culture method	1 × 10^8^ cells/mL	66.08	(125)
*Wickerhamomyces anomalus*	peach	*Monilinia fructigena*, *Monilinia fructicola*	dual culture method		44.12	(52)
					23.53–37.40	

a It is not specified.

**Table 5:** Efficacy of Biocontrol Strategies for Managing Postharvest Pathogens in Fruits Using Yeast

fungal microorganism	fruit	antagonistic yeast	optimum concentration of biocontrol agent	disease incidence (%)	references
*Alternaria alternata*	Pithaya	*Candida inconspicua*, *Pichia kluyveri*	1 × 10^8^ cells/mL	7.4–20.5	(116)
*Aspergillus carbonarius*	grape berries	*Saccharomyces cerevisiae*	1 × 10^6^ cells mL	80	(290)
*Aspergillus niger*	table grape	*Sporidiobolus pararoseus*	1 × 10^9^ cells/mL	75	(126)
*Aspergillus ochraceus*	grape berries	*Saccharomyces cerevisiae*	1 × 10^6^ cells mL	40	(290)
*Aspergillus tubingensis*	grape	*Aureobasidium pullulans*	1 × 10^7^ cells/mL	92.6	(282)
*Botrytis cinerea*	apple	*Hanseniaspora opuntiae*, *Metschnikowia pulcherrima*	1 × 10^7^ cells/mL	14.2	(275)
				6	
*Botrytis cinerea*	strawberry	*Sporidiobolus pararoseus*	1 × 10^8^ cells/mL	20	(291)
*Botrytis cinerea*	grape	*Hanseniaspora uvarum*	1 × 10^8^ cells/mL	51.8	(122)
*Cladosporium cladosporioides*	table grape berries	*Saccharomyces cerevisiae*, *Rhodosporidium paludigenum*, *Rhodosporidium fluviale*, *Wickerhamomyces anomalus*	1 × 10^8^ cells/mL	51.11	(123)
				37.78	
				26.67	
				42.22	
*Colletotrichum gloeosporioides*	ripe olive fruit	*Wickerhamomyces anomalus*	1 × 10^8^ cells/mL	10–40	(292)
*Colletotrichum gloeosporioides*	mango	*Pseudozyma hubeiensis*	1 × 10^8^ cells/mL	49.8	(269)
*Colletotrichum musae*	banana	*Saccharomyces cerevisiae Candida tropicalis*	1 × 10^8^ cells/mL	25	(283)
				25	
*Fusarium oxysporum*	tomato	*Wickerhamomyces anomalus*	1 × 10^6^ cells mL	<50	(286)
*Geotrichum citri-aurantii*	orange	*Saccharomyces cerevisia*, *Candida azyma*, *Rhodotorula mucilaginosa*	1 × 10^7^ cells/mL	38	(293)
				32	
				28	
*Geotrichum citri-aurantii*	citrus	*Metschnikowia citriensis*	1 × 10^8^ cells/mL	30–50	(105)
*Monilinia fructicola*	apple	*Debaryomyces hansenii*, *Wickerhamomyces anomalus*	1 × 10^8^ cells/mL	70.02–92.56	(125)
*Monilinia fructicola*	plums	*Pichia membranaefaciens*, *Kloeckera apiculata*	5 × 10^8^ cells/mL	19.7	(294)
*Monilinia fructigena*	apple	*Rhodoturula glutinis*, *Debaryomyces hansenii*	3 × 10^7^ cells/mL	8.9	(288)
				10.6	
*Penicillium digitatum*	citrus	*Kluyveromyces marxianus*, *Yarrowia lipolytica*	1 × 10^8^ cells/mL	28	(70)
				18	
*Penicillium digitatum*	Mandarin	*Yarrowia lipolytica*	1× 10^7^, 1 × 10^8^ cells/mL	16.67,19.44	(295)
*Penicillium digitatum*	Chongqing Orangery	*Candida fermantati*, *Metschnikowia* sp., *Hanseniaspora uvarum*	1 × 10^8^ cells/mL	6.67	(114)
				16.67	
				66.67	
*Penicillium digitatum*	lemons	*Metschnikowia pulcherrima*	1 × 10^8^ cells/mL	33.3	(86)
*Penicillium digitatum*	oranges	*Rhodotorula mucilaginosa*	1 × 10^8^ cells/mL	31.25	(221)
*Penicillium expansum*	apple	*Meyerozyma guilliermondii*	1 × 10^8^ cells/mL	42.4	(195)
*Penicillium expansum*	table grape	*Aureobasidium pullulans*, *Cryptococcus magnus*, *Metschnikowia pulcherrima*, *Rhodotorula glutinis*	1 × 10^6^ cells mL	33.33–50	(296)
				6.67–50	
				10–50	
				43.33–50	
*Penicillium expansum*	apple	*Kluyveromyces marxianus*	1 × 10^8^ cells/mL	44	(281)
*Penicillium expansum*	pear	*Wickerhamomyces anomalus*	1 × 10^8^ cells/mL	5.56	(297)
*Penicillium expansum*	apple	*Hanseniaspora opuntiae*, *Metschnikowia pulcherrima*	1 × 10^7^ cells/mL	42.1–59.4	(275)
				42.1	
*Penicillium expansum*	table grape berries	*Saccharomyces cerevisiae*, *Rhodosporidium paludigenum*, *Rhodosporidium fluviale*, *Wickerhamomyces anomalus*	1 × 10^8^ cells/mL	83.33	(123)
				73.61	
				77.78	
				90.28	
*Penicillium italicum*	“Valência” sweet orange	*Saccharomyces cerevisiae*	1 × 10^7^ cells/mL	75–92	(265)
*Penicillium italicum*	mandarin	*Meyerozyma guilliermondii*	1 × 10^7^ cells/mL	57.5	(298)
*Penicillium italicum*	mandarin	*Yarrowia lipolytica*	1 × 10^7^ cells/mL, 1 × 10^8^ cells/mL	16.67	(295)
				11.11	
*Penicillium italicum*	leaves, flowers, fruits, and soils	*Saccharomyces cerevisiae*	1 × 10^7^ cells/mL	>80	(265)
*Pestalotiopsis vismiae*	loquat fruit	*Metschnikowia pulcherrima*	1 × 10^8^ cells/mL	43.68	(94)
*Rhizopus stolonifer*	peach	*Pichia guilliermondii*	1 × 10^8^ cells/mL	12.6	(299)

In addition to these studies, the antifungal activity of yeast VOCs was investigated and suggested as a potential biological control technique against *Botrytis cinerea*, *Penicillium expansum*, *Penicillium digitatum*, *Penicillium italicum*, and *Monilinia* sp. with several studies.^52,108−110^ Furthermore, due to the small sizes of these molecules and their diffusion through the environment and soil, yeast VOCs could play a key part in their antagonistic activities.^111^ The antifungal activity of the VOCs of *W. anomalus*, *M. pulcherrima*, and *S. cerevisiae* has been observed in *in vitro* and *in vivo* studies on grapes against *B. cinerea.*(112)

In a recent study by Sansone et al.,^113^ preventive and curative effects of yeast culture on apples were investigated. By the preventive effect, *Rhodosporidium fluviale* severity was reduced to 55% and 75% at the 5^th^ and 10^th^ days of storage with antagonistic yeasts, respectively. However, with the curative effect, the reduction in decay was 48%, which was lower than the preventive effect. To conclude, when the yeast was applied before infection (preventive effect), *Botrytis cinerea* control was more successful than when infection was already present (curative effect). Similar to these studies, higher efficiency has been obtained in preventive than curative activity.^114−116^ As mentioned above, antagonistic yeasts were more effective if they were applied before fungal contamination.

The biocontrol activity of antagonistic yeast increases with higher concentrations, which was also proved by several researchers.^117,118^ For example, 9 log *Cryptococcus albidus* was applied to control *Penicillium expansum* infection on fuji fruit; the spoilage area of the samples reduced to 90.54 or 91.39%, at 95% relative humidity; however, there was no significant impact with the application of 6 logs of yeast.^118^

In another study, Wang et al.^119^ investigated the surface colonization and interaction between *Metschnikowia citriensis* and *Geotrichum citri-aurantii* with SEM studies and observed that fungal pathogens were surrounded by yeast species without deformation. So far, the yeast did not cause any damage to the spores and hyphae but could inhibit the spore germination of *Geotrichum citri-aurantii*. Also, several studies have indicated that spore germination rates were significantly decreased with the yeast treatment, in addition to the reduction of germ tube length.^70,119,120^ Furthermore, the use of autoclaved yeast can evolve into an antifungal biopesticide for managing postharvest fungal rot in pear fruit since it is nontoxic, economical, and environmentally safe. To conclude, yeasts have been identified as possible biocontrol agents because of their minimal nutritional requirements, ability to colonize the surface of fruits quickly, and great stability during storage.

### 3.2 Effects of Antagonistic Yeasts on Host-Fruit Quality

In the case of biocontrol yeast usage, the most critical issue is the harmful effect of the yeast and any fermentation effect in fruit wounds. More than likely, no negative impacts of biocontrol yeasts were observed in any fruit. Several researchers have previously reported that biocontrol yeasts have no effect on quality criteria such as fruit firmness, total soluble solids, and titratable acidity during storage.^116,121−123^ Habiba et al.^124^ found that the firmness of kinnow fruit has been significantly decreased with the extension of the storage period for both nontreated and yeast-treated samples. Additionally, the rise in total soluble solids and the drop in ascorbic acid concentration were lesser in yeast-treated fruits compared to the nontreated kinnow fruit set. For instance, black rot in red pithaya was reduced to 20.5% and 7.4% by *Candida inconspicua* and *Pichia kluyveri*, respectively, after 21 d of storage with antagonistic yeasts, while imazalil-treated fruit diseases have decreased to 47.6%.^116^ The outcomes of these studies demonstrated that a biological agent’s effects depend on storage conditions and the type of fruit.

Also, Czarnecka et al.^125^ reported that in the wounded tissue of apples, POD activity was significantly increased, while CAT activity was decreased during the biocontrol of *Monilinia fructicola* with *Debaryomyces hansenii* and *Wickerhamomyces anomalus* treatment. However, Li et al.^126^ reported that *Sporidiobolus pararoseus* treated table grape enzymatic activities, including PPO, CAT, PAL, and APX, have been increased. Sun et al.^127^ reported that the autoclaved yeast *Rhodosporidium paludigenum* was effective in controlling *Penicillium expansum* in pear fruits. Recent studies also investigated the physicochemical quality of yeast-treated fruits by weight loss, fruit firmness, total soluble solids, titratable acidity, and pH analysis.^116,128^ These studies demonstrated that yeast might prevent fruit dehydration because antagonists decrease deterioration and offer an additional barrier to water diffusion.^116,129^

Meanwhile, the scaled up experiments should be performed to understand the applicability. Therefore, large volumes of yeast biomass must be generated in bioreactors using affordable growth conditions to obtain high yields while maintaining the candidate bioagent’s antagonistic action. For example, postharvest decay of pear by *Penicillium expansum* and *Botrytis cinerea* tried to be controlled by *Pichia membranifaciens* NPCC 1250 and *Vishniacozyma victoriae* NPCC 1263 in two different packing houses and showed a higher reduction in incidence.^130^ In other field trial studies, the yeast treatment’s biocontrol efficiency has been reported to be much more successful than the artificially contaminated and damaged control when mechanical damage was performed.^131^

## 4 Control Approaches to Mitigate Mycotoxin Production

Mycotoxins can be reduced or eliminated by preventing or reducing fungal growth in the field and during postharvest processes. Aflatoxin contamination has been reduced by different yeasts in various crops, including cotton, almond, pistachio, walnut, peanut, and maize.^132^ However, no published information regarding the reduction of aflatoxin by yeasts in fresh fruits and vegetables has been found in the literature.

Although their findings are promising and encouraging, one of the issues studied in less-extend in mycotoxin reduction is using antagonistic yeasts that produce antifungal VOCs, in contrast to mold inhibition studies.^28^*Saccharomyces cerevisiae*, *Candida* sp., and *Kluyveromyces marxianus* VOCs’ ability to reduce mycotoxin production has been recently studied with *in vitro* studies.^81,133,134^ In a study by Galván et al.,^135^ furfuryl acetate (FA) and 2-phenyl ethyl acetate (2PEA) were obtained from *Hanseniaspora uvarum* and *Hanseniaspora opuntiae*. The study’s findings suggest using 2PEA and FA to prevent mycotoxin generation in dried figs during the early postharvest phases. A recent study from Yang et al.^136^ reported that *Meyerozyma guilliermondii* (1 × 10^8^ cells/mL) reduced PAT content by 75% in Shuijing pears at 20 °C for 7 d, demonstrating that PAT could not be effectively controlled at room temperature. Furthermore, the inhibition of *Penicillium expansum* alone was insufficient to remove PAT that was already present in pears. It has also been shown that *Meyerozyma guilliermondi* effectively controlled PAT in Shuijing pear wounds from 3 to 11 d at 4 °C, but the detoxifying mechanism of *Meyerozyma guilliermondii* was not explained in detail. They also stated that the PAT-controlling effect could vary among pear cultivars, and the biological control activity of yeasts depends on concentration. The efficiency of antagonistic yeasts, including *Rhodosporidium kratochvilovae* LS11 and *Cryptococcus laurentii*, combined with a low-dosage synthetic fungicide to control PAT contamination of apples have been studied. According to the results, PAT contamination and fungicide residues have been observed to be lower in apples.^137^ Additionally, *Metschnikowia pulcherrima* reduced *Penicillium expansum* growth and PAT production in apples during storage, while only low but significant reductions have been observed in PAT content,^138^ as shown in Table 6. However, applying *Rhodosporidium paludigenum* at higher concentrations (1 × 10^8^ CFU/mL) increased the PAT accumulation by 24.2 and 12.6 times in apples and pears, respectively, compared with controls.^139^

**Table 6:** Reduction of Mycotoxins by Antagonistic Yeast with *In Vitro* and *In Vivo* Studies

target mycotoxin	antagonistic yeast	mycotoxigenic fungi	medium/host fruit	application dose of antagonistic yeasts	mycotoxin reduction		reference
					*in vitro* application	*in vivo* application	
Aflatoxin B_1_	*Saccharomyces cerevisiae* VOCs	*Aspergillus flavus*	PDA medium	1 × 10^8^ cells/mL	99% reduction	N/A^a^	(81)
Alternaria (Alternariol, alternariol monomethyl ether and tenuazonic acid)	*Hanseniaspora uvarum*	*Alternaria alternata*	synthetic grape medium	b	reduced to 8.5 μg/g from 74.7 μg/g	N/A	(144)
Alternaria (Tenuazonic acid)	*Candida zemplinina*, *Hanseniaspora uvarum*, *Metschnikowia pulcherrima*	*Alternaria alternata*	wine grapes		N/A	81.2–99.8% reduction of TeA production	(143)
Ochratoxin A	*Saccharomyces cerevisiae*	*Aspergillus carbonarius*	synthetic grape medium/CYA medium	1 × 10^6^ cells/mL	reduced to 4217.797 from 1.4327 ng/g	reduced to 1651.20 from 95.63 ng/g	(290)
		*Aspergillus ochraceus*		1 × 10^8^ cells/mL	reduced to 5.6844 from 0.4422 ng/g	N/A	
	*Saccharomyces cerevisiae*	*Aspergillus carbonarius*	grape juice	1 × 10^8^ cells/mL	N/A	reduced to 5 ng/mL from 14.983 and 31.565 ng/mL	(300)
	*Lanchancea thermotolerans*	*Aspergillus section Nigri*	grape	1 × 10^4^-1 × 10^6^ cells/mL	N/A	60–100% reduction	(131)
Patulin	*Pichia caribbica*	*Penicillium expansum*	PDA/apple	5 × 10^8^ cells/mL	58% reduction	reduced to 1.61 from 26.84 μg/mL	(181)
	*Rhodotorula kratochvilovae*	*Penicillium expansum*	Fuji apple	1 × 10^8^ cells/mL	N/A	9.4–16.2% reduction	(301)
	*Metschnikowia pulcherrima*	*Penicillium expansum*	apple	1 × 10^8^ cells/mL	N/A	reduced to 1793.55 μg/fruit in apples from 1995.73 μg/fruit in apples	(138)

a Not applicable.

b It is not specified.

In addition to PAT, several studies have also been conducted to reduce OTA contamination in grape and grape products.^140,141^ The ochratoxigenic species, yeast strains, the water activity, or temperature of the environment, and their interactions affect the inhibitory activities.^140,142^ Prendes et al.^143^ examined the potential of antagonistic yeast to control TeA production in wine grapes. Moreover, in another study, *Metschnikowia* spp., *Hanseniaspora uvarum*, and *Starmerella bacillaris* showed a reduction in mycotoxin (AOH, AME, and TeA) production by *Alternaria alternata. Hanseniaspora uvarum* has been reported as the most effective yeast to diminish mycotoxin production in wine grapes.^144^

## 5 Biodetoxification of Mycotoxins

Biodetoxification of mycotoxins is a relatively new method for the reduction or elimination of mycotoxins by using nonpathogenic microorganisms or their enzymes. Biodetoxification by microorganisms may require the degradation and adsorption of mycotoxins (Figure 3).^145^

### 5.1 Bioadsorption

**Figure 2:**
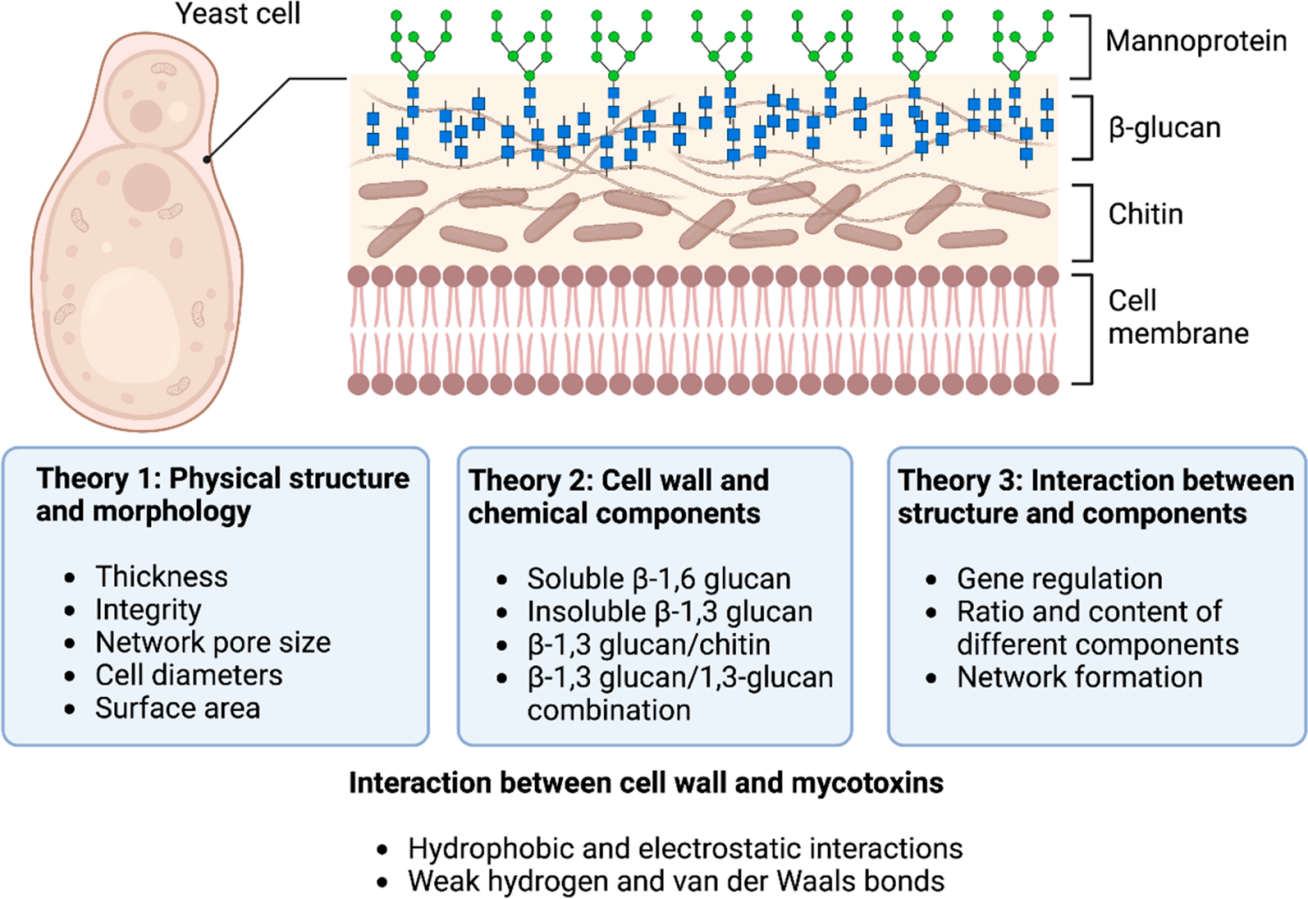
Main theories on yeast cell wall interactions with mycotoxins (adapted from Luo et al.^42^ and Anwar et al.^240^)

Yeasts are promising bioadsorbents for fruit-based mycotoxins (PAT, OTA, AF, CIT, and *Alternaria* toxins).^42^ Mycotoxins can be physically adsorbed by yeast cell wall components containing numerous specific binding sites.^146^ In this regard, glucomannan, mannoprotein, chitin, and β-glucan content in the yeast cell wall can act as bioadsorbents for mycotoxins (Figure 2).^147,148^ Most studies employed heat-inactivated or live yeast cells for the bioadsorption of mycotoxins (Table 7).

**Table 7:** Bio-adsorption Methods of Yeasts for Mycotoxin Removal in Fruits, Fruit Juices, and Culture Media

target mycotoxin	antagonistic yeast	fruit/fruit-based food matrix/culture media	mycotoxin adsorbance	reference
Aflatoxin B_1_, Aflatoxin B_2_	*Kluyveromyces lactis* + *Saccharomyces cerevisiae* ATCC 64712	PBS	65.84% in 72 h	(302)
			64.52% in 72 h	
Aflatoxin B_1_	*Saccharomyces cerevisiae* PTCC 5052	H_2_O + MeOH (60:40)	84% in 12 h	(303)
Alternariol, alternariol methyl ether	*Saccharomyces cerevisiae* GIM 2.169	aqueous solution	>85% in 60 h	(187)
Citrinin	*Saccharomyces cerevisiae* PTCC 5052 + *Monascus purpureus* ATCC 16362	aqueous solution	79.7% in 13 d	(184)
Ochratoxin A	*Candida intermedia*	grape juice	83% in 48 h	(160)
Patulin	*Candida tropicalis*	kiwi fruit juice	90% in 24 h	(146)
Patulin	*Saccharomyces cerevisiae* YS-3	apple juice	>60% in 10 h	(155)
			>80% in 24 h	
Patulin	*Saccharomyces cerevisiae* YS-3	acetic acid aqueous solution and apple juice	2.69 mg/g in 70 h	(304)
Patulin	*Saccharomyces cerevisiae* CCTCC 93161	apple juice	100% in 48 h	(153)

Dietary exposure to mycotoxins can be reduced by the ability of yeasts to bind to mycotoxins.^149^ In this respect, alive and dead *Saccharomyces cerevisiae* cells were used effectively in mycotoxin adsorption.^148,150^ In the study of Guo et al.,^151^ laboratory-prepared (LYP) and commercial yeast powder (CYP) samples (inactivated *S. cerevisiae* yeasts) were used to adsorb PAT in apple juice without deteriorating the juice’s quality. Following 48 h of incubation at 29 °C, PAT removal was higher at pH 5.0 for both LYP and CYP, with reduction rates of 73.66% and 83.12%, respectively. Moreover, Yue et al.^152^ added inactive yeast powders (10 different *S. cerevisiae* strains) to remove PAT from apple juice. The study’s findings showed that more than 50% of PAT was eliminated from apple juice in just 24 h, with >72% being the highest reduction rate. Besides, Zhang et al.^153^ reported that *S. cerevisiae* CCTCC 93161 adsorbed 85.88% and 100% of PAT (500 μg/L) from apple juice at 30 °C for 24 and 48 h, respectively. Additional investigation into the PAT removal process found that proteins and polysaccharides on the yeast surface played a key role in bioadsorption.

Furthermore, *S. cerevisiae* and *Saccharomyces bayanus* dead (heated) cells successfully decontaminated 90% of OTA from grape juice within 5 min for 72 h of incubation.^154^*S. cerevisiae* YS-3 was also reported to adsorb >60% and >80% of OTA in apple juice in 10 and 24 h, respectively.^155^*S. cerevisiae* strains were also investigated for their OTA adsorption capabilities under mimicked gastrointestinal conditions. The results showed that a probiotic yeast *S. cerevisiae* var. *boulardii* ATCC MYA-796 (10^7^ CFU/mL) adsorbed over 44% of OTA (100 μg/L) within 1 h. However, mycotoxin binding is somewhat reversible, and the net amount of OTA binding was discovered to be about 21%. To develop functional foods, foods can be enriched with OTA-adsorbing yeasts, allowing these beneficial microorganisms to bind to OTA in the gastrointestinal tract.^156^

Furthermore, yeasts belonging to *Pichia* spp., *Phaffia* spp., *Rhodotorula* spp., *Schizosaccharomyces* spp., *Cryptococcus* spp., *Candida* spp., and *Kloeckera* spp. have adsorption activity on mycotoxins.^42,157,158^ For instance, *Candida tropicalis* N-10 adsorbed 75.1% of PAT (200 μg/L) within 30 h in kiwi fruit juice with a minimum effect on the juice’s flavor. They also emphasized that the mycotoxin removal was linked with yeast cell surface morphology.^159^

The study by Farbo et al.^160^ tested *Candida intermedia* to detoxify OTA in grape juice resulting in 83% removal of OTA in 48 h. In addition, Campagnollo et al.^161^ investigated yeast-based products for their ability to bind AFs. The yeast strain, contact time, pH, and temperature influenced AFs adsorption. Among the yeasts tested, *Zygosaccharomyces rouxii* and *Cyberlindnera fabianii* were found to have the highest and the lowest adsorption capacities with 86.4% and 18.45%, respectively. Depending on the strain of interest, the physicochemical conditions, structure, and concentration of yeast cells and toxins affect mycotoxin binding.^43^ In light of these studies, it can be concluded that inactivated yeast cells showed better mycotoxin adsorption than that of live yeast cells.^157^

### 5.2 Biodegradation

Mycotoxin biodegradation is an environmentally friendly and effective control strategy.^158^ Yeasts hold great potential in altering fruit-based mycotoxins (PAT, OTA, AF, CIT, and *Alternaria* toxins) into non- or less toxic derivatives.^162^ Antagonistic yeasts play a significant role through their biological (intracellular/extracellular) enzymes and cellular metabolism.^163−165^

Current research on mycotoxin detoxification by antagonistic yeasts in fruits has focused on PAT and OTA, with little attention paid to CIT in fruits, which was also linked to PAT and OTA occurrence.^4^ PAT is the most commonly reported mycotoxin in fruit-derived products, and its removal was found to be concentration-dependent.^166−168^ When the concentration of PAT increased from 10 to 100 mg/L, the removal rate of PAT by *Rhodotorula mucilaginosa* decreased to half, indicating that the initial mycotoxin concentration was a determining factor.^169^ However, as the PAT concentration increases, the bioprotective yeasts’ multiplication rate may decline. For example, the growth of *Candida guilliermondii* was slightly restrained at 100 μg/mL of PAT concentration, and it decreased and continued growing (8 × 10^8^ cells/mL) at 500 μg/mL for a 120 h of incubation causing PAT reduction. Similarly, *Metschnikowia pulcherrima’*s growth was slightly inhibited in the presence of PAT in the culture medium compared to control,^138^ suggesting that antagonistic yeasts need to be screened for PAT resistance before use in the biodegradation process.^170^

In a recent research, Fu et al.^163^ demonstrated that PAT-induced viable *Meyerozyma guilliermondii* cells completely biodegraded PAT via intracellular enzymes, known as short-chain dehydrogenase (Table 8). In this line, Xing et al.^171^ cloned and purified a short-chain dehydrogenase/reductase from *Candida guilliermondii* to biodegrade PAT. This PAT-degrading enzyme (150 μg/mL) was employed to reduce 80% of PAT into *E*-ascladiol in apple juice without affecting the juice quality. Likewise, Orotate phosphoribosyltransferase (0.15 g/L) from *Rhodotorula mucilaginosa* was used to remove 80% of patulin (1 mg/L) from apple juice at 25 °C for 18 h.^172^ Furthermore, fractions of *Pichia caribbica*’s intracellular enzymes digested PAT into both *E*- and *Z*-ascladiol, showing that multiple enzymes in yeasts may be involved in the degradation.^173^

**Table 8:** Bio-degradation Methods of Yeasts for Mycotoxin Removal in Fruits, Fruit Juices, and Culture Media

antagonistic yeast	fruit/fruit-based food matrix /culture media	application dose	mycotoxin concentration	mycotoxin reduction (%)		reference
				*in vitro* application	*in vivo* application	
Citrinin						
*Cryptococcus podzolicus* Y3	nutrient yeast dextrose agar	1 × 10^8^ cells/L	20 μg/mL	98% in 42 h	N/A^a^	(305)
Ochratoxin A						
*Cryptococcus podzolicus* Y3	grape juice/PM medium (maltose, sucrose, tryptone, and yeast extract)	1 × 10^8^ cells/L	1 μg/mL	100% in 5 d	100% in 3 d	(306)
*Yarrowia lipolytica*	nutrient yeast dextrose agar	1 × 10^8^ cells/L	0.1 μg/mL	88% in 24 h	N/A	(307)
Patulin						
*Rhodotorula mucilaginosa*		>1 × 10^8^ cells/L	10 μg/mL	<90% at 35 °C in 21 h	N/A	(169)
*Pichia guilliermondii* S15–8	apple juice simulated solution	1 × 10^8^ cells/L	1 mg/L	>90% at 30 °C in 24 h	N/A	(168)
*Metschnikowia pulcherrima* Y29	peptone malt extract liquid medium	1 × 10^8^ cells/L	1000 μg/L	∼50% at 25 °C	N/A	(138)
*Meyerozyma guilliermondii*	nutrient yeast dextrose agar	1 × 10^8^ cells/L	10 μg/mL	93% in 48 h	N/A	(163)
*Saccharomyces cerevisiae*	yeast extract, peptone, glucose liquid medium	1 × 10^8^ cells/L	20 μg/mL	100% at 28 °C in 5 d.	N/A	(162)
*Cryptococcus laurentii*, *Kosakonia radicincitans*, *Cryptococcus laurentii* + *Kosakonia radicincitans*	apples	1 × 10^8^ cells/L (1:1, v/v)	5000 μg/L	N/A	45.30% at 25 °C for 10 d	(308)
					30.31% at 25 °C for 10 d	
					100% at 25 °C for 10 d	
*Rhodotorula mucilaginosa*	NYDB medium	1 × 10^8^ cells/L	10 μg/mL	100% in 24 h	N/A	(309)

a Not applicable.

Likewise, *Metschnikowia pulcherrima* was used to control *Penicillium expansum* and PAT accumulation in apples, which completely removed PAT in a culture medium at 25 °C after 120 h.^138^ However, there was no PAT in the yeast cell wall or its intracellular metabolites, indicating that PAT was transformed into an unidentified molecule. Although chromatographic and spectroscopic techniques (e.g., NMR, LC-MS/MS, and GC-MS/MS) can detect mycotoxin byproducts, some of them may be undetectable, highlighting the need for novel analytical techniques.^43,167^ It is challenging to remove PAT during the processing of apple products because it is particularly stable in acidic environments; nevertheless, yeast enzymes and metabolites are promising in PAT removal.^162^

*Rhodotorula kratochvilovae* LS11^174^ and *Rhodotorula mucilaginosa*(169) detoxified PAT into desoxypatulinic acid, *Pichia guilliermondii* S15–8,^168^ and *Candida guillierondii*(170) transformed PAT into *E*-ascladiol, and *Saccharomyces cerevisiae*(175) and *Pichia caribbica*(176) completely degraded PAT into *Z*-ascladiol and *E*-ascladiol by intracellular enzymes. During apple cider brewing, Wang et al.^177^ demonstrated that *Saccharomyces cerevisiae* 1027 could effectively biodegrade PAT into the less-toxic compound *E*-ascladiol by intracellular enzymes, which played a greater role than extracellular enzymes.

A recent study by Zhang et al.^178^ investigated the *Meyerozyma guilliermondii*’s PAT stress on enzymes and the regulatory role of related transcription factors. In addition to conventional extraction and purification of enzymes, genetic engineering tools can enable the recombinant production of these mycotoxin-degrading enzymes more affordably. With these tools, it is also possible to produce several enzymes to degrade multiple mycotoxins simultaneously.^167,179^ Accordingly, a tailored microbial consortium of specific species or strains can precisely biotransform multiple mycotoxins into non-, less-, or more toxic compounds.^42,167^ For example, a study by Ma et al.^180^ showed that PAT-induced intracellular enzymes of *Hannaella sinensis* completely reduced PAT in a pear juice medium within 42 h. In another study, *Pichia caribbica* (5 × 10^8^ cells/mL) and PAT-producing *P. expansum* (5 × 10^4^ spores/mL) were inoculated into apple wounds. After incubation at 20 °C for 15 days, PAT content was reduced (94%) to 1.61 μg/mL with respect to control (26.84 μg/mL).^181^

Although the mechanisms for the elimination of PAT have been partially clarified, a thorough understanding of the process is still needed to be provided at the molecular level. Regarding OTA, *Cryptococcus podzolicus* Y3 was shown to completely degrade it into a less-toxic compound OTα via its intracellular enzymes in both aqueous media in 5 d and grape juice in 3 d. Moreover, *Cryptococcus podzolicus* Y3 degraded OTA and CIT simultaneously in an aqueous solution, however, the degradation efficiency was lower than that of a single mycotoxin.^182^ Recently, the same research group examined the whole-genome sequencing of *Cryptococcus podzolicus* Y3 to unveil the OTA toxicity and detoxification mechanisms during OTA degradation.^183^ More recently, Wei et al.^158^ cocultured the antagonistic yeast *Cryptococcus podzolicus* Y3 with *N*-acetyl-l-cysteine (NAC) to enhance OTA degradation. Combining *C. podzolicus* Y3 with 10 mM NAC has transformed OTA into less toxic OTα, resulting in 100% and 92.6% of OTA removal within 1 and 2 d, respectively.

In another study, pigment-negative and CIT-producing *Monascus purpureus* and the alive yeasts *Saccharomyces cerevisiae* were cocultured for 3 d to enhance pigment production and CIT reduction. Subsequently, CIT was adsorbed by *S. cerevisiae* by 79.7%, and the production of *M. purpureus* pigments was increased. The hydrolytic enzymes of *S*. *cerevisiae* were thought to disrupt the cell wall of *Monascus purpureus*, causing pigment leakage and stimulating pigment production by its metabolites.^184^ Likewise, Patharajan et al.^185^ tested three yeast strains (*Metschnikowia pulcherrima* MACH1, *Pichia guilliemondii* M8, and *Rhodococcus erythropolis* AR14) for their OTA degradation capabilities. The results indicated that the MACH1 strain degraded more than 80% of OTA in 15 d at 30 °C, while other yeasts degraded over 50%. The researchers attempted to understand the degradation mechanism. The principal process for the biodegradation of OTA involves either the hydrolysis of the lactone ring or the hydrolysis of the amide bond between the iso-coumarin residue and phenylalanine. OTα is considered nontoxic or less hazardous than the documented breakdown products.^186^ However, neither cell wall adsorption nor the production of byproducts (e.g., OTα and phenylalanine) was detected, suggesting that existing methods are insufficient.

Besides, using inactivated *Saccharomyces cerevisiae* powder eliminated the *Alternaria* mycotoxins (AOH and AME) from the aqueous solution. The yeast cell components were thought to be involved in the adsorption mechanism.^187^ In some cases, mycotoxins can form conjugates with the food matrix (e.g., proteins and polysaccharides), referring to masked mycotoxins. These mycotoxins can escape detection using routine analytical methods, resulting in an underestimation of mycotoxin exposure and risk.^188^ Inside the gastrointestinal tract, these hidden fungal toxins can be digested and released into their free forms, posing a threat to health. The masked mycotoxins were mostly reported in cereals.^189^ This concept could also be used for fruit juices or other fruit-based products. In this regard, Soukup et al.^190^ first reported the glycosylated conjugates of AOH and AME in tomato fruit. These substances can be called masked mycotoxins and may release their conjugated molecules into the human digestive tract, posing a health hazard.

## 6 Combined Application of Antagonistic Yeasts for Enhanced Biocontrol Efficacy

Studies have also been conducted to see if the combinations of antagonistic yeasts with different agents or processes enhance their performance since the inconsistent efficiency of antagonistic yeasts remains one of the challenging issues that must be addressed. While studies indicate that their biocontrol efficiency may be increased, limited studies examine the combined effect on the antimycotoxigenic characteristics of yeasts. There are several studies combining agents, such as β-glucan,^191,192^ phytic acid,^193^ methyl jasmonate,^194,195^ melatonin,^196^*N*-acetylglucosamine,^197^ γ-aminobutyric acid,^198^ cinnamic acid,^199^ glycine betaine,^182,200,201^ chitosan,^202,203^ calcium chloride,^204^ and arginine.^205^ Apart from the combination with an agent, the impact of different processes on the efficiency of the antagonistic yeast was also investigated, including hot air,^206^ microwave,^207^ UV-C irradiation,^208−211^ and heat shock.^212^ When these agents or processes are combined with antagonistic yeasts, their biocontrolling activity against phytopathogenic/mycotoxigenic fungi is enhanced, potentially inhibiting mycotoxins.

### 6.1 Combination with an Agent

There may be different mechanisms behind the increase in the efficiency of antagonistic yeasts, but the number of studies examining this is limited. β-glucan, a natural polysaccharide found in the cell walls of many cereals and microorganisms, plays an important role in the yeast cell wall by increasing yeast resistance to stress and thereby contributing to the enhancement of antagonistic activity.^213^ In the study of Wang et al.,^214^ the application of antagonistic yeast with β-glucan on apple wounds induced enzymes involved in the development of disease resistance and the reduction of oxidative damage. The role of phytic acid combined with yeasts was found to be significant for apples^193^ and strawberries to decrease spoilage according to phytic acid concentration^215^ (Table 9). It is also known that various extracts of natural origin also have antimicrobial effects and can increase the efficiency of antagonistic yeasts, such as cardoon leaf extract combined with *Wickerhamomyces anomalus* BS91.^216^ In the study applying *Adansonia digitata* L. (Baobab) extract together with *Sporidiobolus pararoseus* Y16 to increase the yeast’s activity against *Penicillium expansum,* no correlation between the disease incidence value and the concentration of the agent was found.^217^

**Table 9:** Agents and Processes to Enhance the Biocontrol Efficiency of Antagonistic Yeasts

combination		yeast	pathogen	fruit	storage conditions	decay incidence (%)			reference
						control^a^	yeast	combination	
agent	compound class								
*Adansonia digitata* L. (Baobab)	plant extract	*Sporidiobolus pararoseus* Y16	*Penicillium expansum*	apple	20 °C, 95% RH^b^	100%	∼^c^60%	∼40%	(217)
alginate oligosaccharide	bioactive compound	*Meyerozyma guilliermondii*	*Penicillium italicum*	Mandarin	25 °C, 4 d	100%	88.3%	36.7%	(298)
		*Debaryomyces hansenii*	*Penicillium expansum*	apple	20 °C, 95% RH, 5 d	100%	∼35–40%	15.28%	(310)
Ascorbic acid	water soluble vitamin	*Pichia caribbica*	*Penicillium expansum*	apple	20 °C, 5 d	100%	∼25%	10.37%	(223)
					25 °C, 95% RH, 15 d	100%	38.9%	19.4%	(89)
β-aminobutyric acid	nonprotein amino acid	*Hanseniaspora uvarum*	*Botrytis cinerea, Alternaria alternata*	kiwifruit	25 °C, 4 d	100%	45–53%	∼30–35%	(93)
β-glucan	polysaccharide	*Cryptococcus podzolicus*	*Penicillium expansum*	pear	20 °C, 95% RH, 4 d	100%	52.77%	30.53%	(213)
		*Cryptococcus podzolicus*	*Penicillium expansum*	apple	20 °C, 5 d	100%	∼70%	40.88%	(214)
calcium chloride	inorganic compound	*Meyerozyma guilliermondii* YS-1, *Meyerozyma caribbica* YS-3, *Cryptococcus albidus* YS-4, *Cryptococcus* sp. YS-5	*Penicillium expansum*	apple	21 °C, 7 d	100%	100%	75–80%	(311)
carboxymethyl chitosan	chitosan derivative, polysaccharide	*Cryptococcus laurentii*	*Penicillium italicum*	grapefruit	22 °C, 85–95%, 4 d	73.33%	∼45–50%	36.66%	(312)
chitosan	polysaccharide	*Pichia anomala*	*Penicillium expansum*	grape	20 °C, 95% RH, 5 d	100%	∼23%	∼15%	(202)
cinnamic acid	organic compound	*Cryptococcus laurentii*	*Penicillium italicum*	mandarin	25 °C, 95% RH, 4 d	100%	∼50%	∼20%	(199)
methyl jasmonate	volatile organic compound	*Cryptococcus laurentii*	*Penicillium digitatum*	mandarin	20 °C, 4 d	100%	∼80%	40.3%	(194)
		*Meyerozyma guilliermondii*	*Penicillium expansum*	apple	20 °C, 95% RH	100%	42.4%	21.6%	(195)
γ-aminobutyric acid	nonprotein amino acid	*Sporidiobolus pararoseus* Y16	*Aspergillus tubingensis*	grape	20 °C, 70–75% RH, 5 d	100%	52.78%	16.67%	(198)
glycine betaine	organic osmolytes	*Pichia caribbica*	*Penicillium expansum*	apple	25 °C, 95% RH, 15 d	91.66%	48.81%	32.14%	(182)
		*Sporidiobolus pararoseus* Y16	*Penicillium expansum*	apple	20 °C, 90% RH, 7 d	100%	d	11.1%	(200)
phosphatidylcholine	soybean extract	*Hanseniaspora uvarum* Y3	*Penicillium digitatum*	orange	20 °C, 95% RH, 7 d	∼100%	∼30–35%	∼5–6%	(313)
phytic acid	antioxidant	*Pichia caribbica*	*Penicillium expansum*	apple	20 °C, 95% RH, 10 d	100%	∼75%	∼30%	(193)
		*Rhodotorula mucilaginosa*	*Botrytis cinerea*	strawberry	20 °C, 95% RH, 3 d	100%	∼50%	∼25–55%	(215)
salicylic acid	phenolic compound	*Pichia membranaefaciens*	*Penicillium digitatum*, *Penicillium italicum*	*Citrus sinensis* L.	20 °C, 85–90% RH, 6 d	100%	88.89%	77.56%	(222)
							83.33%	72.22%	
sodium bicarbonate	chemical compound	*Kluyveromyces marxianus*	*Penicillium digitatum*	Mandarin	3–6 d	100%	41.67–70%	18.33–58.33%	(314)
trehalose	glucose derivative	*Sporidiobolus pararoseus* Y16	*Penicillium expansum*, *Aspergillus tubingensis*	grape	20 °C, 6 d	100%	∼25–30%	∼15–20%	(315)
oligochitosan	oligosaccharide	*Pichia caribbica*	*Alternaria alternata*	tomato fruit	20 °C, 90% RH, 4 d	∼85%	∼20%	∼5–20%	(226)
oligogalacturonide	oligosaccharide	*Candida oleophila*	*Botrytis cinerea*, *Alternaria alternata*	kiwifruit	25 °C, 4 d	100%	51%	33%	(277)
process	condition								
hot water treatment	45 °C, 5 min	*Aureobasidium pullulans* L1, L8 and *Trichoderma harzianum* Th1	*Neofabraea vagabunda* ID02	apple in two ripening classes	20 °C, 21 d	untreated ∼ 60–80%	∼5–10%	complete inhibition	(237)
	53 °C, 2 min	*Pichia membranaefaciens*	*Penicillium digitatum*, *Penicillium italicum*	*Citrus sinensis* L.	20 °C, 8 d	untreated ∼ 100%	∼100%	88.89%	(316)
								83.33%	
microwave	2450 MHz, 2 min	*Metschnikowia pulcherrima*	*Penicillium citrinum*	Jujube	25 °C, 5 d	100%	36%	21%	(207)
UV-C irradiation	4 kJ/m^2,^ 12 min	*Cryptococcus laurentii*	*Botrytis cinerea*, *Alternaria alternata*	tomato fruit	20 °C, 4 d	untreated 100%	∼40–50%	∼24–30%	(211)
	5 kJ/m^2,^ 15 min	*Metschnikowia pulcherrima*	*Alternaria alternata*	Jujube	22 °C, 7 d	100%	∼20–30%	∼15–20%	(317)
	4 kJ/m^2,^ 12 min	*Pichia cecembensis*	*Fusarium oxysporum*, *Alternaria alternata*	melon	25 °C, 5 d	untreated 100%	∼40–50%	∼20–25%	(208)

a Sterile distilled water.

b Relative humidity.

c About.

d Unexamined.

Salicylic acid is a phenolic component found in plants and emerged as an alternative to manage fungal diseases. Lyousfi et al.^218^ showed a significant effect in combination. Coqueiro et al.^219^ observed the molecular and genetic changes caused by the applied agents in food. Calcium chloride alone does not affect *Colletotrichum musae*, the cause of crown rot on bananas, whereas the antagonistic activity of yeast significantly increased in combination.^220^ Salicylic acid was thought to be involved in the defense mechanisms such as an increase in the amount of lignin, which is an important mechanism against fungal infections,^221^ and an increase in enzyme activity when combined with *Hanseniaspora uvarum*.^122^ However, the implication in combination is critical because salicylic acid applied alone to citrus fruit has no effect, whereas when combined with antagonistic yeast, it has an antagonistic effect.^222^ In another study investigating the mechanism, 250 μg/mL of ascorbic acid with *Pichia caribbica* yeast showed the lowest decay incidence, and some of the expressed proteins decreased while others increased. In particular, the expression of genes associated with some enzymes was increased, and it was stated that these genes were involved in the biocontrol of antagonistic yeasts.^223^ Similarly, ascorbic acid was more effective with *Pichia caribbica* and improved oxidative stress tolerance and antioxidant enzyme activities.^89^ Additionally, gamma and beta aminobutyric acid compounds, which are classified as different nonproteinogenic amino acids, were applied to the fruits in combination with yeasts,^93,198^ it is important to examine the increase in antagonistic activity at the disease incidence level. It is expected that the yeast population will increase as the yeast adaptation to the environment increases. According to Cheng et al.,^93^ since no adverse effects were observed in the yeast population, the beta aminobutyric acid and yeast combination can be employed to manage postharvest diseases.

In the studies, almost all agents used for combination were preferred because of their protective role on foods, inducing various enzyme activities or indirectly affecting nutrients. For instance, methyl jasmonate, a plant-derived volatile compound known as an endogenous phytohormone,^224^ increased the activity of enzymes (POD, PPO, PAL, and CAT) that are important for treating blue mold disease.^195^ In addition to the effect on the enzyme, the effect on antioxidant activity was also shown for Chinese bayberries.^225^ Additionally, enzymes responsible for the defense mechanism of *Kluyveromyces marxianus* were found to be improved when combined with *N*-acetylglucosamine.^197^ However, the antagonistic activities of yeasts have been associated with compounds such as pulcherrimin. Therefore, promoting pulcherrimin production will lead to increased antagonistic activity.^205^

Presently, consumers tend to use natural products, therefore, combining antagonistic yeasts with natural and safe agents appears to be an important parameter in biocontrol.^203^ Chitosan has been indicated as an alternative polymer among the components to be combined, and it enhanced the blue mold disease inhibition ability of *Pichia anomala* in grapes.^202^ In another study, oligochitosan combined with *Pichia caribbica* positively affected the ROS mechanism and the enzymes active in this mechanism. The implementation of combined treatment increased fruit resistance by decreasing the disease-causing components in the fruit.^226^ It was determined that the combination of *Pichia caribbica* with bamboo leaf flavonoid prevented the growth of *Penicillium expansum* and reduced the amount of PAT in *in vitro* analyses.^227^ The effectiveness of the agent used may vary depending on the product, the target microorganism, and storage conditions such as temperature.

### 6.2 Combination with Process

Apart from agents, different physical methods may be applied with antagonistic yeasts to extend the shelf life of fruits and vegetables and reduce losses due to postharvest mold-related diseases.^228^ UV-C irradiation is one of the methods applied to fruit and has also been tested for preventing postharvest fungal diseases.^229^ According to a recent study, it helps to improve the postharvest quality of fruit by boosting the activity of antioxidant enzymes and reducing mycotoxins.^230^ Additionally, combination studies with yeasts were also available to demonstrate the method’s efficacy when applied alone. Likewise, the study of Zhang et al.^211^ reported that the enzyme production was enhanced, and UV-C irradiation had no effect on the growth of *Cryptococcus laurentii* yeast applied for biocontrol purposes. *Pichia cecembensis*, one of the antagonistic yeasts used for biocontrol, significantly contributed to the control of postharvest decay in melons when combined with UV-C.^208^ Increased enzyme activity and improved antioxidant properties contributed to the effectiveness of the combination of UV-C irradiation and *Candida tropicalis* against fungal decay in pineapple.^210^ Besides, it was aimed to prevent the undesirable changes caused by *Penicillium citrinum* in jujube fruit by combining *Metschnikowia pulcherrima* and microwave application.^207^ As in other studies, this combination produced more effective results than the applications conducted alone. Terao et al.^231^ combined hot water brushing and UV-C irradiation methods with *Candida membranifaciens* CMAA-1112 antagonistic yeast as postharvest treatment. It was observed that the combination of process and yeast had no adverse effect and showed an additive effect to control green mold on orange. In UV-C and yeast treatment, as in other studies, the change in enzyme activities was also analyzed since it is an important criterion. Activity changes of PAL, GLU, and CHT enzymes were analyzed and UV-C treatment with *Candida tropicalis* was presented as an alternative to extending the shelf life of pineapple.^210^ The modified atmosphere packaging (MAP) method, which is one of the important food preservation methods, controlled fungal growth in apples by applying antagonistic yeasts depending on the selected gas composition.^232^ Another study found that combining MAP and yeasts on sweet cherries increased the product’s shelf life and delayed the appearance of fungal disease effects. de Paiva et al.^233^ and Zhao et al.^234^ treated cherry tomatoes with hot air at 38 °C in combination with *Pichia guilliermondii* and obtained more effective results contrary to single applications. There may be different mechanisms behind this increased effect; for example, in the study of Wei et al.,^235^ hot air treatment affected defense mechanisms in cherry tomato fruit, especially phenylpropanoid metabolisms, which affect phenolics and flavonoids. Additionally, thermal shock treatment has also been shown to increase the antagonistic activity of yeasts. Cheng et al.^236^ documented that thermal shock treatment increased the antagonistic activity of *Rhodotorula mucilaginosa* and improved its stress tolerance; comparable results were also obtained in a study with *Metschnikowia fructicola*.^212^ Ultrasound and ohmic heating were also mentioned as alternative physical application methods.^228^ Furthermore, different antagonistic yeasts combined with hot water treatment showed a remarkable effect by completely inhibiting the growth of *Neofabraea vagabunda* in apples.^237^ Similarly, Zhou et al.^222^ showed the effectiveness of hot water treatment in combination with antagonistic yeast to manage green and blue mold disease. While the treatment did not affect the growth of antagonistic yeast at different storage temperatures, it was interpreted that the hot water treatment had an effect, especially on nutrient and space competition, and the higher efficiency of the combination was attributed to this mechanism. Additionally, the activities of different enzymes, especially those responsible for the defense mechanism, were increased. However, it has been shown that the biocontrol activities of antagonistic yeasts change in the presence of temperature stress.^238^ Therefore, when antagonistic yeasts are combined with hot applications, it is important to how the procedure is carried out. Another application method of antagonistic yeasts is the production of fruit-coating materials containing biocontrol agents. Apple coating material integrated with *Metschnikowia pulcherrima* was produced and observations made during storage showed that the coating with the biocontrol agent increased the capacity of antagonistic yeast to colonize and was more effective in terms of antifungal activity. In addition, it was shown that the application also has antimycotoxigenic effects.^239^

As a result, combinations with antagonistic yeasts may be used to reduce fungi-induced postharvest diseases in fruits to the maximum extent possible. In this way, it will be possible to reduce not only food loss, but also the usage of chemicals. However, although various antagonistic mechanisms have been examined, further studies are required on the causes of inducing the mechanism. Alternative methods may be offered by applying different combinations to different fruits.

## 7 Future Prospects

Growing public awareness and tighter regulations have led to trends toward biosafe, ecological, and cost-effective strategies to inhibit postharvest diseases in fruits and fruit-based products caused by pathogenic and/or mycotoxigenic fungi, as well as to decrease mycotoxins. Following these purposes, research in this field has focused on antagonistic yeasts and their enzymes due to their high adsorption and biodegradation capabilities on mycotoxins. The current literature findings are related to the use of antagonistic yeasts in the postharvest treatments of fruit-related fungi and their mycotoxins, as well as the biodetoxification capabilities of yeasts on fruit-based mycotoxins were discussed. Antagonistic yeasts used for biocontrolling purposes are on the GRAS list, and their toxicity analyses need to be performed prior to approval. Because of this, harnessing antagonistic yeasts to protect fruits and fruit products from fungal infestations and mycotoxins while also improving quality and shelf life is deemed safe. Most antimycotoxigenic studies were carried out on patulin and ochratoxin in fruit and fruit-derived products. Antagonistic yeasts show promise in the biodetoxification of fruit-based products. In some cases, biodegradation products may be more toxic than the initial mycotoxin. Sometimes these toxic molecules can avoid detection using existing techniques, demonstrating the need for new analytical methods. Further research is required on the biochemical basis of detoxification pathways and evaluating detoxification byproducts.

Antagonistic yeasts may exhibit inconsistent biocontrolling efficacy. To overcome this issue, they can be used in collaboration with other yeasts, bacteria, bioagents, and physical processes. However, yeasts must be checked before applying these combinations to verify their viability at the application concentration. In this way, these integrated disease management approaches may act synergistically or additively to increase biocontrolling activity. Understanding the mechanisms of biological control action is critical to the development of rational combinations of selected fruits or antagonistic consortia. However, there are limited studies regarding how a tailored microbial consortium will prevent fungi proliferation and decompose mycotoxins, in which food matrix, at what level, and the conditions. As a result, future approaches may rely on the combinations of different microorganisms to provide additional benefits in mycotoxin degradation and adsorption.

Furthermore, climate change has reduced crop yields and increased multimycotoxin formation. Good Agricultural Practice and Good Manufacturing Practice applications can be implemented in this regard during the postharvest stage. In addition, multiple mycotoxins in the food matrix can be precisely removed using specialized microbial consortia and their metabolites. In this line, the development of genetic engineering could make it possible to generate mycotoxin-degrading enzymes or enzyme cocktails effectively and inexpensively, enabling the simultaneous degradation of several mycotoxins. Moreover, this enzyme preparation would prevent food products from undergoing unnecessary further processing. The main challenge lies in moving these strategies from the laboratory to the field and adapting them to large-scale commercial applications.

**Figure 3:**
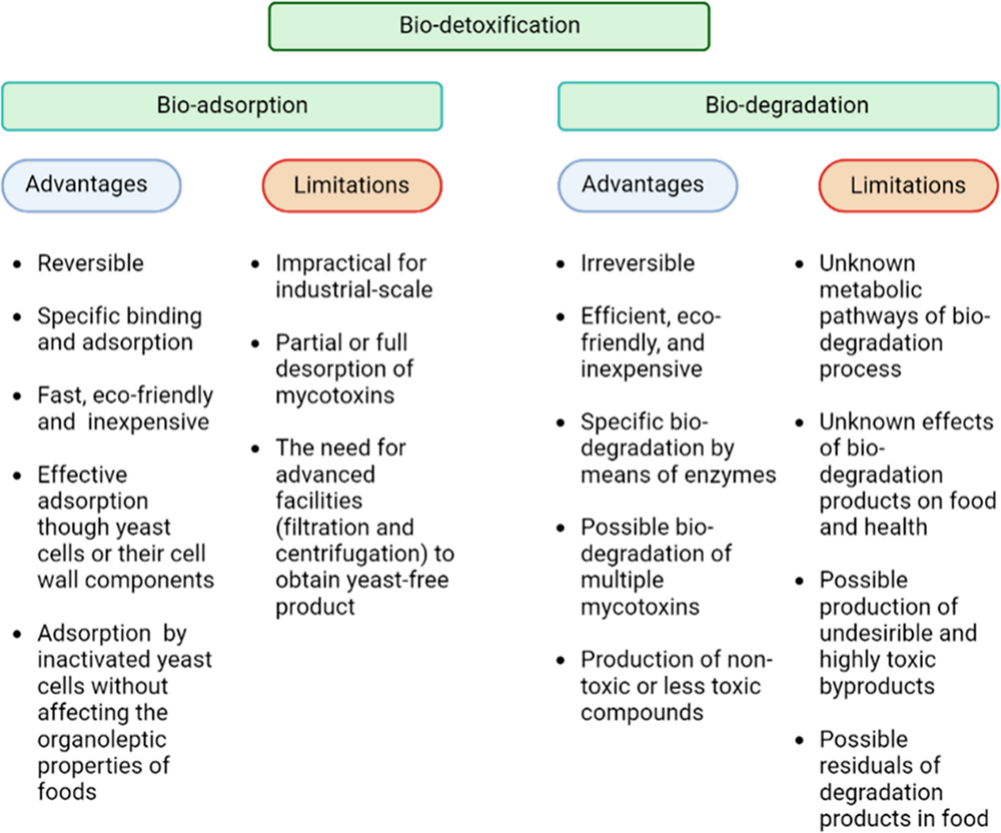
Advantages and limitations of biological methods on mycotoxins (adapted from Yang et al.^241^).
